# Epidemiology of Acute Respiratory Infections in Children in Guangzhou: A Three-Year Study

**DOI:** 10.1371/journal.pone.0096674

**Published:** 2014-05-05

**Authors:** Wen Kuan Liu, Qian Liu, De Hui Chen, Huan Xi Liang, Xiao Kai Chen, Mei Xin Chen, Shu Yan Qiu, Zi Yeng Yang, Rong Zhou

**Affiliations:** 1 State Key Laboratory of Respiratory Diseases, National Clinical Research Center for Respiratory Disease, Guangzhou Medical University, Guangzhou, Guangdong, China; 2 The First Affiliated Hospital of Guangzhou Medical University, Guangzhou, Guangdong, China; University Hospital San Giovanni Battista di Torino, Italy

## Abstract

Acute Respiratory Infections (ARI) are some of the most common human diseases worldwide. However, they have a complex and diverse etiology, and the characteristics of the pathogens involved in respiratory infections in developing countries are not well understood. In this work, we analyzed the characteristics of 17 common respiratory pathogens in children (≤14 years old) with ARI in Guangzhou, southern China over a 3-year period using real-time polymerase chain reaction. Pathogens were identified in 2361/4242 (55.7%) patients, and the positivity rate varied seasonally. Ten of the 17 pathogens investigated showed positivity rates of more than 5%. The most frequently detected pathogens were respiratory syncytial virus (768/2361, 32.5%), influenza A virus (428/2361, 18.1%), enterovirus (138/2361, 13.3%), *Mycoplasma pneumoniae* (267/2361, 11.3%) and adenovirus (213/2361, 9.0%). Co-pathogens were common and found in 503 of 2361 (21.3%) positive samples. When ranked according to frequency of occurrence, the pattern of co-pathogens was similar to that of the primary pathogens, with the exception of human bocavirus, human coronavirus and human metapneumovirus. Significant differences were found in age prevalence in 10 of the 17 pathogens (p≤0.009): four basic patterns were observed, A: detection rates increased with age, B: detection rates declined with age, C: the detection rate showed distinct peaks or D: numbers of patients were too low to detect a trend or showed no significant difference among age groups (p>0.05). These data will be useful for planning vaccine research and control strategies and for studies predicting pathogen prevalence.

## Introduction

Acute respiratory infections (ARI) result in the death of an estimated 4 to 5 million children each year in developing countries [1]–[4]. Most of these deaths are among children with pneumonia. Respiratory tract infection etiology is complex and diverse. In developed counties, the major causes of ARI in children and adults are influenza A and B viruses (infA, infB), parainfluenza virus type 1 (PIV1), PIV2, PIV3, respiratory syncytial virus (RSV), adenovirus (ADV) and rhinovirus [5]–[6]. However, there is a lack of data on the characteristics of ARI in developing nations. Over the past decade, a number of new pathogens have been reported, including human metapneumovirus (HMPV) [7] and human bocaviurs (HBoV) [8], thus increasing the urgency for the study of epidemiology of respiratory tract pathogen infections in developing countries.

Over the past two decades, virus isolation and serology have been the mainstay of clinical laboratory diagnosis for respiratory virus infections. The introduction of molecular-based detection methods has made diagnosis quicker and cheaper and increased the ability to detect more than one virus simultaneously. In this work, the epidemiological features of 17 respiratory pathogens in children with ARI were studied in Guangzhou, southern China. This study should serve as a valuable resource for information on ARI and provide useful data for future research and development of vaccines.

## Materials and Methods

### Ethics Statement

The study was approved by The First Affiliated Hospital of Guangzhou Medical University Ethics Committee for research on human beings, and all participants or their guardians gave signed informed consent for participation in the study.

### Respiratory Sample Collection

Pediatric patients (≤14 years old) who presented with at least two of the following symptoms: cough, pharyngeal discomfort, nasal obstruction, snivel, sneeze, dyspnea or who were diagnosed with pneumonia by chest radiography during the previous week, were enrolled in this study. Chest radiography was conducted according to the clinical situation of the patient, and pneumonia was defined as an acute illness with radiographic pulmonary shadowing (at least segmental or in one lobe) by chest radiography.

Throat swab samples were collected from the enrolled patients at three hospitals in Guangzhou, southern China between July 2009 and June 2012. The samples were refrigerated at 2 to 8°C in viral transport medium, transported on ice to State Key Laboratory of Respiratory Diseases and analyzed immediately or stored at −80°C before testing.

### Real-time Polymerase Chain Reaction (PCR) for Detection of Respiratory Tract Pathogens

Each sample was tested simultaneously for the following 17 respiratory tract pathogens: influenza A virus (infA), influenza B virus (infB), four types of parainfluezna (PIV1, PIV2, PIV3, PIV4), respiratory syncytial virus (RSV), adenovirus (ADV), enterovirus (EV), human metapneumovirus (HMPV), four strains of human coronavirus (HCoV-229E, OC43, NL63 and HKU1), human bocavirus (HBoV), *Mycoplasma pneumoniae* (MP), and *Chlamydophila pneumoniae* (CP). The testing procedure has been described in previous reports [9]–[10].

### Statistical Analysis

Statistical analysis was performed using SPSS statistical software (version 19.0; SPSS Inc., Chicago, IL, USA). For comparisons of categorical data, the χ^2^ test and Fisher’s exact test were used as appropriate. All tests were two-tailed and p<0.05 was considered statistically significant.

## Results

Samples from a total of 4242 pediatric patients were analyzed over three years from July 2009 to June 2012 in Guangzhou, southern China. Male to female ratio of the patients was 1.92∶1. Median age was 1.5 years (interquartile range, 0.67 to 3.00), with a range from one day to 14 years old, and with 3713/4242 (87.5%) patients being less than 5 years old. Pathogens were detected in 2361/4242 (55.7%) patients with a median age of 1.42 years (interquartile range, 0.67 to 3.0). Male to female ratio was 2.04∶1 (p = 0.076) in the positive patients and 1.82∶1 in the negative patients.

**Figure 1:**
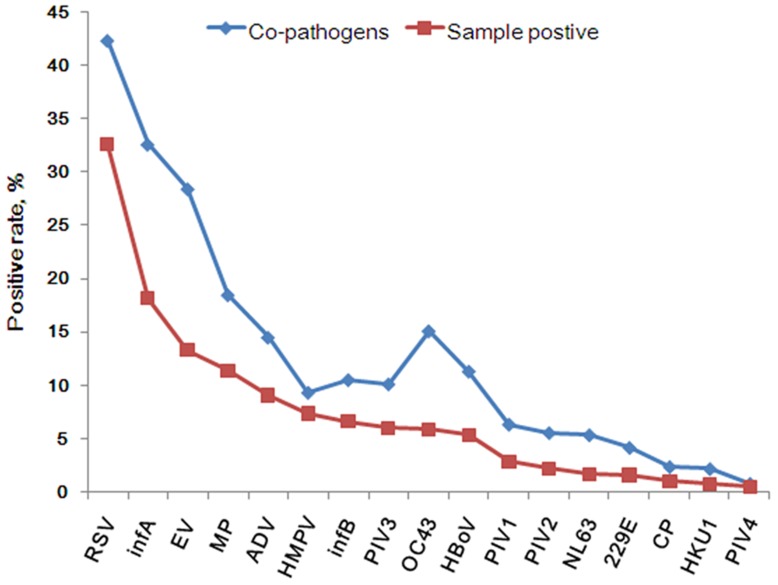
The rank of pathogen detection rate and co-pathogen rate in 4242 pediatric patients with ARI in Guangzhou from July 2009 to June 2012. 229E: human coronavirus 229E, OC43: human coronavirus OC43, NL63: human coronavirus NL63, HKU1: human coronavirus HKU1.

The most frequently detected pathogens in this study were RSV (768/2361, 32.5%), infA (428/2361, 18.1%), EV (138/2361, 13.3%), MP (267/2361, 11.3%) and ADV (213/2361, 9.0%), followed by HMPV, infB, PIV3, HCoV-OC43, HBoV all with the detection rates higher than 5.0%. The positivity rates of the remaining seven pathogens were lower than 5.0% (Table 1). Co-pathogens were common and found in 503 of 2361 (21.3%) positive-samples (Table 1). The frequency of detection of the co-pathogens followed almost the same ranking order as that of the primary pathogens with the exception of HBoV, HCoV-OC43 and HMPV (Figure 1).

**Table 1:** Detection of respiratory tract pathogens from 4242 pediatric patients with ARI by real-time PCR.

Pathogens#	infA	infB	RSV	EV	ADV	PIV1	PIV2	PIV3	PIV4	229E	OC43	NL63	HKU1	HBoV	HMPV	MP	CP	Detection rate in 2361positive samples, %
**infA**	**428**	16	63	32	12	6	4	7	0	5	18	4	2	7	7	19	0	18.1
**infB**		**155**	13	9	1	0	1	5	0	0	1	0	1	2	5	5	2	6.6
**RSV**			**768**	63	21	7	12	10	1	8	19	10	1	17	8	16	5	32.5
**EV**				**313**	16	12	1	10	1	3	9	5	0	10	10	10	2	13.3
**ADV**					**213**	1	3	3	1	3	4	5	1	7	5	10	2	9.0
**PIV1**						**67**	2	1	0	0	4	1	0	3	0	6	0	2.8
**PIV2**							**51**	3	0	2	8	0	0	1	1	2	0	2.2
**PIV3**								**140**	0	0	9	1	0	6	2	9	1	5.9
**PIV4**									**11**	0	0	0	0	0	0	1	0	0.5
**229E**										**37**	5	2	0	0	2	1	0	1.6
**OC43**											**138**	2	0	4	6	11	2	5.8
**NL63**												**39**	1	1	1	1	1	1.7
**HKU1**													**17**	3	0	5	0	0.7
**HBoV**														**125**	3	10	0	5.3
**HMPV**															**173**	4	0	7.3
**MP**																**267**	1	11.3
**CP**																	**23**	1.0
**Single pathogen**	264	102	555	170	140	35	23	89	7	16	62	12	6	68	126	174	11	78.7
**Co-pathogens**	164	53	213	143	73	32	28	51	4	21	76	27	11	57	47	93	12	21.3

# 229E: human coronavirus 229E, OC43: human coronavirus OC43, NL63: human coronavirus NL63, HKU1: human coronavirus HKU1.

*Boldface indicates total numbers of pathogens detected. Note: multiple pathogens were isolated from some samples.

**Figure 2:**
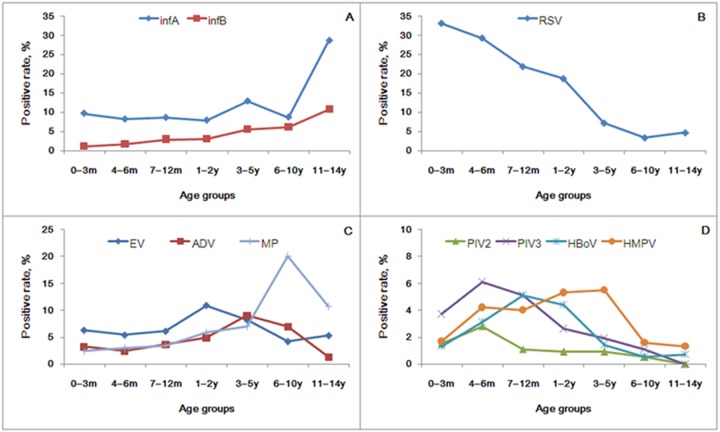
The patterns of pathogen distribution among different pediatric age groups in 4242 pediatric patients with ARI in Guangzhou from July 2009 to June 2012. 229E: human coronavirus 229E, OC43: human coronavirus OC43, NL63: human coronavirus NL63, HKU1: human coronavirus HKU1. A: Detection rates increased as age increased (p<0.001); B: Detection rates declined as age increased (p<0.001); C and D: Detection rate peak occurred as age increased (p≤0.009).

In this study, patients were divided by age into seven groups. There was no difference in pathogen positivity rate (p = 0.338) and co-pathogen detection rate (p = 0.117) among the age groups. However, significant differences were found in age prevalence between infA, infB, RSV, EV, ADV, PIV2, PIV3, HBoV, HMPV, MP infections (p≤0.009, Table 2). In general, four age/prevalence patterns could be distinguished among children in this study; A: Detection rates increased with age as illustrated in infA and infB (Figure 2A); B: Detection rates declined with age, as shown in RSV (Figure 2B); C: The detection rates peaked at different ages as in EV (peak age 1–2 years, 10.9%), ADV (peak age 3–5 years, 9.0%), PIV2 (peak age 4–6 months, 2.8%), PIV3 (peak age 4–6 months, 6.1%), HBoV (peak age 7–12 months, 5.1%), HMPV (peak age 3–5 years, 5.5%) and MP (peak age 6–10 years, 20.1%) (Figure 2C and 2D) and D: the number of samples was too small to analyze or no significant pattern was present (p>0.05), as in PIV1, PIV4, HCoV-OC43, −229E, -NL63, -HKU1 and CP.

**Table 2:** Distribution of pathogen-positive patients among different age groups.

Pathogen#	0–3 m (n = 463) *	4–6 m (n = 575)	7–12 m (n = 822)	1–2 y (n = 997)	3–5 y (n = 856)	6–10 y (n = 379)	11–14 y (n = 150)	p value
**Positive samples**	259 (55.9)	327 (56.9)	473 (57.5)	563 (56.5)	458 (53.5)	194 (51.2)	87 (58)	0.338
**Co-pathogens**	66 (14.3)	79 (13.7)	94 (11.4)	126 (12.6)	88 (10.3)	36 (9.5)	14 (9.3)	0.117
**infA**	45 (9.7)	47 (8.2)	71 (8.6)	79 (7.9)	110 (12.9)	33 (8.7)	43 (28.7)	**<0.001**
**infB**	5 (1.1)	10 (1.7)	24 (2.9)	30 (3)	47 (5.5)	23 (6.1)	16 (10.7)	**<0.001**
**RSV**	153 (33)	168 (29.2)	179 (21.8)	186 (18.7)	62 (7.2)	13 (3.4)	7 (4.7)	**<0.001**
**EV**	29 (6.3)	31 (5.4)	50 (6.1)	109 (10.9)	70 (8.2)	16 (4.2)	8 (5.3)	**<0.001**
**ADV**	15 (3.2)	14 (2.4)	30 (3.6)	49 (4.9)	77 (9)	26 (6.9)	2 (1.3)	**<0.001**
**PIV1**	4 (0.9)	10 (1.7)	21 (2.6)	14 (1.4)	12 (1.4)	5 (1.3)	1 (0.7)	0.239
**PIV2**	7 (1.5)	16 (2.8)	9 (1.1)	9 (0.9)	8 (0.9)	2 (0.5)	0 (0)	**0.009**
**PIV3**	17 (3.7)	35 (6.1)	42 (5.1)	26 (2.6)	16 (1.9)	4 (1.1)	0 (0)	**<0.001**
**PIV4** 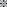	0 (0)	2 (0.3)	0 (0)	4 (0.4)	4 (0.5)	1 (0.3)	0 (0)	–
**229E** 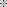	5 (1.1)	4 (0.7)	9 (1.1)	10 (1)	6 (0.7)	3 (0.8)	0 (0)	–
**OC43**	9 (1.9)	17 (3)	33 (4)	43 (4.3)	18 (2.1)	13 (3.4)	5 (3.3)	0.077
**NL63** 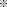	5 (1.1)	6 (1)	10 (1.2)	5 (0.5)	7 (0.8)	5 (1.3)	1 (0.7)	–
**HKU1** 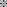	1 (0.2)	0 (0)	5 (0.6)	3 (0.3)	6 (0.7)	1 (0.3)	1 (0.7)	–
**HBoV**	6 (1.3)	18 (3.1)	42 (5.1)	44 (4.4)	12 (1.4)	2 (0.5)	1 (0.7)	**<0.001**
**HMPV**	8 (1.7)	24 (4.2)	33 (4)	53 (5.3)	47 (5.5)	6 (1.6)	2 (1.3)	**0.001**
**MP**	11 (2.4)	17 (3)	28 (3.4)	59 (5.9)	60 (7)	76 (20.1)	16 (10.7)	**<0.001**
**CP** 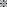	15 (3.2)	0 (0)	3 (0.4)	1 (0.1)	0 (0)	4 (1.1)	0 (0)	**–**

No. (%) of each group except where specifically stated.

# 229E: human coronavirus 229E, OC43: human coronavirus OC43, NL63: human coronavirus NL63, HKU1: human coronavirus HKU1.

*m: month(s); y: year(s).

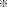
Not done because of the small number of positive samples obtained.

**Figure 3:**
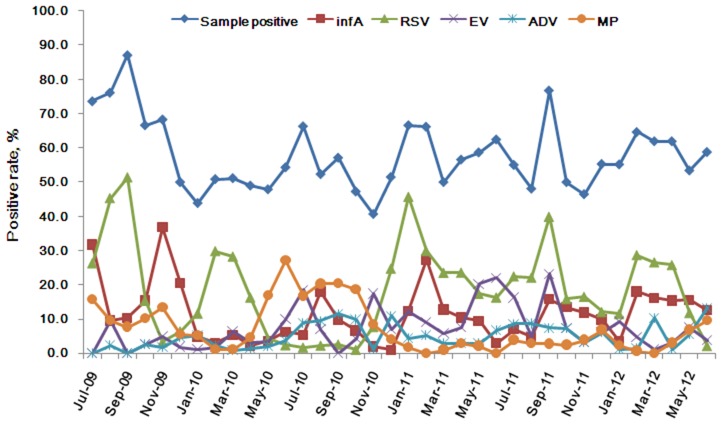
Monthly distribution of the pathogen-positive samples and the most frequency pathogens in 4242 pediatric patients with ARI in Guangzhou from July 2009 to June 2012.

**Figure 4:**
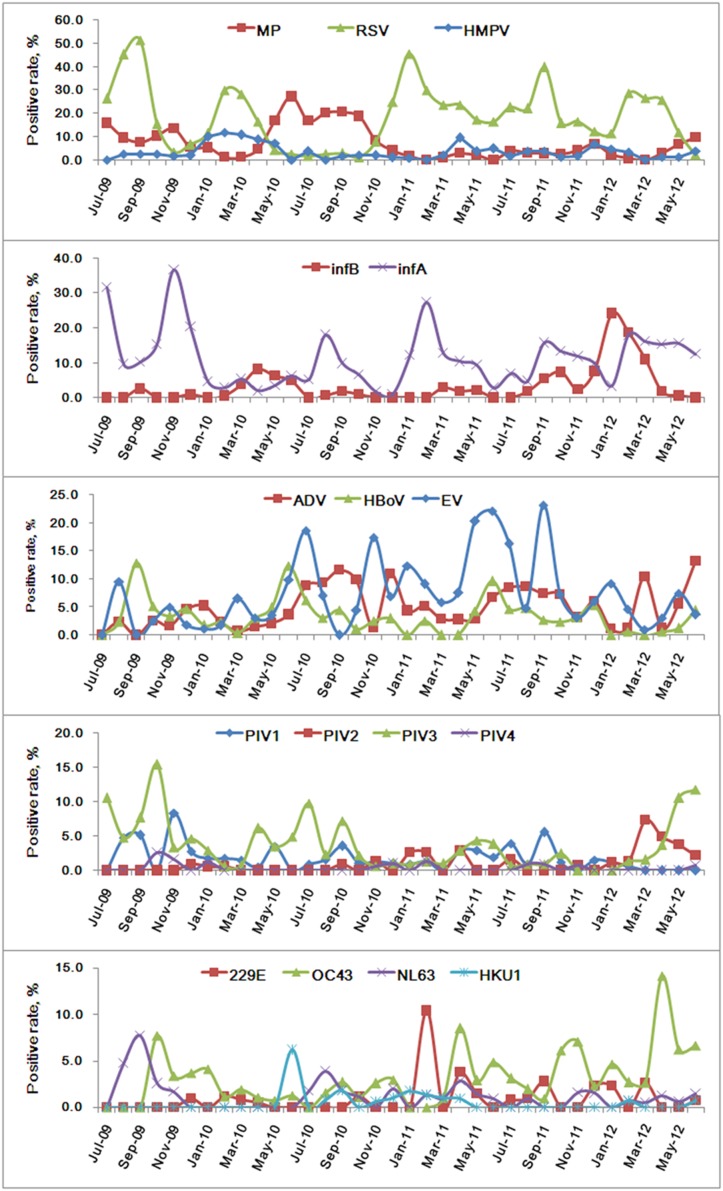
Seasonal distribution of the pathogens in 4242 pediatric patients with ARI in Guangzhou from July 2009 to June 2012. CP isolates were not included in the graph due to few positive cases. 229E: human coronavirus 229E, OC43: human coronavirus OC43, NL63: human coronavirus NL63, HKU1: human coronavirus HKU1.

In general, sample positivity rates increased when seasons changed as is shown in Figure 3. In this study, RSV, infA, EV, MP and ADV formed the bulk of the positive sample (Figure 3). In seasonal distribution, RSV and infA, PIV, and human coronavirus infection mainly occurred at the change from winter to spring and summer to autumn; MP and infB increased as RSV and infA declined, respectively. ADV mainly occurred in summer and autumn, although it was generally distributed all year round; HMPV occurred at the change of season from spring to summer and from winter to spring; EV and HBoV occurred mostly in summer and HBoV was prevalent in winter (Figure 4). Few CP positive samples were found, and so a seasonal pattern could not be determined.

## Discussion

ARI is one of the most common human diseases, and the heaviest burden of viral respiratory illness is carried by children. Studies using viral culture diagnosis have estimated that, in developed countries, infants and preschool children experience a mean of 6–10 viral infections annually and school-age children and adolescents experience 3–5 illnesses annually [11]–[12]. RSV, infA and infB, PIV1, 2, and 3, and ADV are considered to be the most common causative viruses for ARI, especially for lower respiratory tract illness [5]–[6], [13]. MP and CP are the causative agent of atypical pneumonia and have been studied widely in developed countries. However, the characteristics of respiratory pathogens in the etiology of ARI in developing countries are not well studied. In this work, we analyzed the characteristics of 17 common respiratory pathogens from throat swab samples which were collected from children with ARI in Guangzhou, southern China over a 3-year period. Although the best recovered samples should be bronchial alveolar lavage fluid (BALF) in the location of pulmonary infection. However, it is hardly carried out in clinic for harvesting BALF in illness kids. Throat swab is practically clinical behavior for taking the specimen in patients with ARI so far.

Among pediatric patients with ARI in this study, 55.7% (2361/4242) patients were positive for one or more of the 17 pathogens studied, and the positivity rate would have been higher if rhinovirus and bacteria had been included. 10 of the 17 pathogens showed positivity rates greater than 5%, demonstrating the wide diversity of pathogens contributing to ARI (Figure 1; Table 1). In general, the distributions of pathogens vary among different countries and regions because of climatic and other variables. In southern China, RSV, infA, EV, MP, ADV were the predominant pathogens in this study and thus were the major influence on the structure of our analysis (Figure 3). Seasonal distributions of most of the pathogens rose during the change of seasons (Figure 4).

RSV (32.5%, 768/2361) was the most frequently isolated virus occurring mainly among children less than 2 years old. This distribution is consistent with previous reports of RSV in both developed and developing countries [13]–[18] (Table 1, Table 2). RSV is known to occur in well-defined recurrent epidemics during the cold season in temperate climates [6], [19]. In tropical and subtropical areas, RSV infections have been reported to peak more often in the wet season, but locations close to the equator show a less consistent pattern, some with almost continuous RSV activity and with varying seasonal peaks [19], [21]. In this study, two seasonal peaks of RSV were found at the changes of season from winter to spring and from summer to autumn. This pattern is similar to that reported in Nepal [18].

Influenza virus is one of the major causative agents of respiratory disease in humans, and leads to a more severe disease than the common cold which is caused by a different type of virus [23]. Influenza occurs worldwide with outbreaks during the winter season in temperate countries [24]–[25]. In this study, infA (428/2361, 18.1%) was the second most frequent pathogen-isolated, and showed a seasonal distribution that was similar to RSV, except for the period of the pandemic of H1N1 in 2009 and 2010 [26]–[28] (Table 1, Figure 4). infB occurred less often than infA and was the sixth most frequent pathogen found.

EV, MP and ADV were also important pathogens in our study. EV is frequently isolated from the throats of people with respiratory infection and many studies have supported the role of EV in ARI [29]–[30]. In a seven-year study in Rio de Janeiro EV was found less frequently than RSV, ADV and influenza viruses in patients with ARI [31].

MP is the causative agent of atypical pneumonia and is also responsible for other respiratory tract infections such as tracheobronchitis, bronchiolitis, croup, and less severe upper respiratory tract infections in older children and young adults [32]. Epidemics generally occur at intervals of 4–7 years [33]. In our study, MP was the fourth most frequent pathogen isolated and tended to increase when RSV was declining (Figure 4), in contrast with a previous report [34]. However, both pathogens could be found all year long.

ADV infection usually causes symptoms of low respiratory illness (LRI) in children, and can occur all year round. In Guangzhou, ADV was mainly found in summer and autumn, but was also present all year long (Figure 4).

PIVs are important causes of ARI, especially in children [35]–[36]. An estimated five million LRI occur each year in the United States among children under 5 years old, and PIVs have been isolated in up to one third of these infections [37]–[39]. However, a total of 11.4% (269/2361) of patients in our study were PIV positive, and PIV3 which is the most frequent PIV isolated was found in only 5.9% of patients, which is lower than that found in the United States study.

In the past decade, at least six new viruses associated with respiratory infection have been identified, including HMPV, severe acute respiratory syndrome coronavirus, human coronavirus NL63 and HKU1, PIV4, and HBoV [5], [41], demonstrating the diversity of ARI pathogens. Many viruses like rhinovirus and human coronavirus have been largely ignored by the medical community because their clinical impact was considered to be minor. It is now clear that these viruses, once thought to cause only a common cold, can also cause pneumonia in adults [42]. All these viruses are common causes of sporadic cases or outbreaks of community-acquired infections and can be fatal in immunosuppressed patients and the elderly [43]. Thus, there is still much work that needs to be done in characterizing the effects of these new pathogens.

Over the past two decades, virus isolation and serology have been the mainstay of clinical laboratory diagnosis for respiratory virus infections. Because of the limited sensitivity of these methods, co-pathogen rates have generally been low. As expected, with the development of new diagnostic methods based on real-time PCR many more co-pathogens are being detected. Co-pathogen infections were found in 503 of the 2361 (21.3%) positive patients, and the frequency of the causative pathogens was similar to that among the primary pathogens with the exception of HBoV, HCoV-OC43 and HMPV (Figure 1), with HMPV occurring less as a co-infection than HBoV and HCoV-OC43. The clinical relevance of detection of co-pathogens in pneumonia, and the association with severe illness, is uncertain [44]–[47]. Viral-viral interaction *in vivo* is poorly understood. Viruses might interact indirectly or directly with each other, resulting in complementation or inhibition. In one study, children with pneumonia caused by co-pathogenic infection with HBoV and other viruses suffered more wheezing than those with viral pneumonia associated with only one pathogen [48].

In general, ARI occurs mostly in children under the age of 5 [6], [47]. In our study, 87.5% (3713/4242) of the patients were less than 5 years old. No significant difference was found in positivity rate (p = 0.338) and co-pathogen rate (p = 0.117) among different age groups (Table 2). However, most pathogens showed significant differences in age prevalence with the exception of PIV1 (p = 0.239), HCoV-OC43 (p = 0.077), and pathogens for which there were too few positive samples to analyze such as PIV4, 229E, NL63, HKU1 and CP. Four age group distribution patterns could be observed as shown in Figure 2. In general, influenza virus (infA and infB) increased with age, while RSV declined as age increased (Figure 2A and 2B). The attack rate of influenza virus was typically highest in school-age children and daycare populations as has been shown in previous reports [49]–[52]. This probably represents the higher intensity of transmission behavior in this population coupled with a relatively low rate of immunity. RSV is a cause of pediatric hospital admissions for LRI in most areas of the world. Most severe disease occurs in children aged under one year, with a peak occurring between 2 and 5 months of age, as indicated previously [52]–[54]. In addition, evidence suggests that RSV LRIs are associated with persistent diminished airway size in part owing to altered regulation of airway tone.

Significant differences in age distribution were seen in influenza and RSV (p<0.001), also in seven other pathogens EV, ADV, PIV2, PIV3, HBoV, HMPV and MP (p≤0.009) (Table 2), and different age groups showed different distribution peaks (Figure 2). This was consistent with previous serologic and epidemiologic studies of these pathogens [20], [22], [31], [33], [40], [55]–[57]. For the seven remaining pathogens no statistical differences in age distribution or few positive samples were found to analyze.

ARI is a complex and diverse group of diseases; it is difficult to identify the pathogen from clinical symptoms. Despite many advances, further studies are still needed to better understand the role of different pathogens in the cause and pathogenesis of ARI, especially in developing countries. The data from this work might be useful for advance warning of ARI and for development of effective vaccines.
